# Preventing Impaired Driving

**Published:** 1999

**Authors:** Ralph W. Hingson, Timothy Heeren, Michael R. Winter

**Affiliations:** Ralph W. Hingson, Sc.D., is a professor and chair of the Social and Behavioral Sciences Department; Timothy Heeren, Ph.D., is a professor in the Epidemiology and Biostatistics Department; and Michael R. Winter, M.P.H., is a statistical coordinator in the Data Coordinating Center at the Boston University School of Public Health, Boston

**Keywords:** moderate AOD use, drinking and driving, AOD impairment, BAC, DWI laws, sanction, AOD availability, minimum drinking age, traffic accident, AODR (alcohol and other drug related) mortality, literature review

## Abstract

Although moderate drinking does not necessarily increase a person’s blood alcohol concentration (BAC) to the level at which driving is legally prohibited in the United States, any drinking can impair driving tasks. In addition to laws establishing lower legal BAC limits for drivers, legislative approaches for reducing alcohol-impaired driving include imposing sanctions for drinking and driving and restricting alcohol’s availability.

A driver does not necessarily have to be intoxicated to be impaired by alcohol. Even moderate drinking, defined as drinking no more than two drinks per day for men and no more than one drink per day for women (National Institute on Alcohol Abuse and Alcoholism [NIAAA] 1995), may impair driving performance. This level of drinking generally results in a blood alcohol concentration (BAC) of 0.03 percent for both men and women (if the drinks are consumed in 1 hour on an empty stomach) (see figure 1). A person’s risk of involvement in a fatal crash nearly doubles with each 0.02-percent increase in BAC (Zador 1991). In 1997, 18 percent of fatally injured drivers who tested positive for alcohol had BACs between 0.01 and 0.09 percent (National Highway Traffic Safety Administration [NHTSA] 1998*a*), a range that falls below the legal BAC limit for drivers in most States. Eight percent of these drivers had BACs between 0.01 and 0.04 percent.

This article explores the relationship between alcohol consumption and impairment and examines legislative approaches for reducing alcohol-impaired driving, including laws lowering the legal BAC limits for drivers, sanctions imposed for impaired driving, and strategies for restricting alcohol’s availability.

## Moderate Drinking and Impairment

Even at low BAC levels, alcohol impairs driving performance by reducing the driver’s reaction time and slowing his or her decisionmaking process (Moskowitz et al. 1985). A driver’s ability to divide his or her attention between two or more visual stimuli can be impaired at BACs of 0.02 percent or lower (Starmer 1989; Howat et al. 1991; Moskowitz et al. 1985). Starting at BACs of 0.05 percent, drivers exhibit impairment in eye movement, glare resistance, visual perception, reaction time, certain types of steering tasks, information processing, and other driving components (Starmer 1989; Howat et al. 1991; Hindmarch et al. 1992; Finnegan and Hammersley 1992). Currently, the legal BAC limit for noncommercial drivers in most States is 0.10 percent. Thus, although moderate drinking may impair driving ability, most States have legal BAC limits for drivers that exceed the BAC level reached as a result of moderate drinking. Consequently, moderate drinkers, although often impaired, can still drive legally.

**Figure 1:**
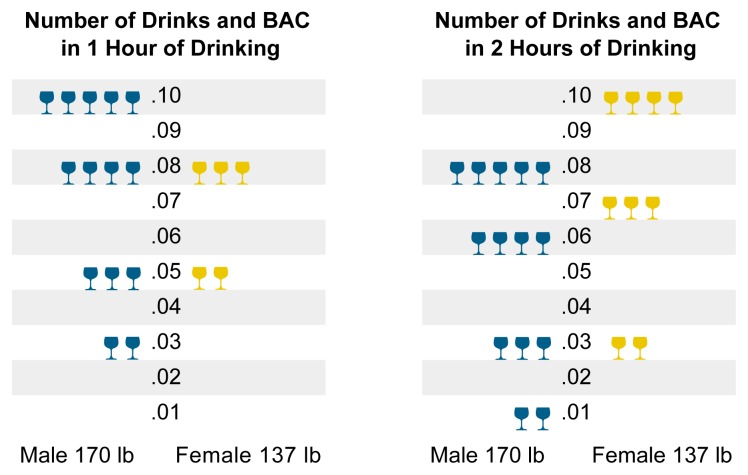
Average blood alcohol concentration (BAC) after different levels of alcohol consumption. In this diagram, one drink equals 0.54 oz alcohol; this is the approximate amount found in one shot of distilled spirits, one can of beer, or one glass of wine. 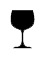
 = one drink; lb = pound; oz = ounce.

Alcohol absorption and metabolism vary among people, depending on such factors as drinking pace, food consumption, age, gender, and the proportion of body mass that is fatty tissue. Typically, a 170-pound man would need to consume five drinks in 1 hour on an empty stomach to reach a BAC of 0.10 percent. To reach a BAC of 0.08 percent, the legal limit in 17 States, a 170-pound man would need to consume four drinks in 1 hour on an empty stomach (see figure 1).

Women absorb and metabolize alcohol differently than do men. In general, compared with men, women contain a smaller amount of body water to absorb each drink. Women also exhibit lower activity levels of the alcohol-metabolizing enzyme alcohol dehydrogenase (ADH) in the stomach, causing a larger portion of ingested alcohol to reach the blood (NIAAA 1997). A 137-pound woman would need to consume three drinks in 1 hour on an empty stomach to reach a 0.08-percent BAC and four drinks in 2 hours to reach a 0.10-percent BAC (see figure 1). Drinking over a longer period of time and eating while drinking extend the number of drinks required to reach these BAC levels (NHTSA 1997).

Although moderate drinking may not cause a person’s BAC to exceed the legal limit for driving, moderate drinking increases the risk of being involved in a fatal crash. Compared with drivers who have not consumed alcohol, drivers with BACs between 0.02 and 0.04 percent are 1.4 times as likely to be involved in a single-vehicle fatal crash. Furthermore, this risk increases to an estimated 11.1 times higher for drivers with BACs between 0.05 and 0.09 percent, 48 times higher for drivers with BACs between 0.10 and 0.14 percent, and 380 times higher for drivers with BACs at or above 0.15 percent (Zador 1991).

For drivers under age 21, fatal crash risk increases more with each 0.02-percent increase in BAC than it does for older drivers (Zador 1991). At all BAC levels including zero, the fatal crash risk for female drivers ages 16 to 20 is at least double the risk for female drivers age 25 and over, and the risk for male drivers ages 16 to 20 is triple the risk for male drivers age 25 and over (Zador 1991). Young drivers generally have less driving experience than older drivers and are more likely to take risks in traffic, such as speeding, disobeying traffic signals, and not wearing safety belts (Hingson and Howland 1993). Because alcohol consumption further increases the risk of crash involvement for young drivers, all States have adopted zero-tolerance laws for drivers under age 21, prohibiting driving after any alcohol consumption.

Recognizing the threat to the public safety associated with even moderate drinking and driving by transportation workers, the Federal government prohibits commercial truck drivers, railroad and mass transit workers, marine employees, and aircraft pilots from operating their vehicles with a BAC at or greater than 0.04 percent. To reach this relatively low BAC limit, however, most people would have to drink above the level of moderate drinking. The American Medical Association (1986) has endorsed lowering the legal BAC limit to 0.05 percent for all drivers; however, no State has yet adopted this standard.

Several other countries’ legal BAC limits for drivers come closer than the United States to restricting drivers to moderate drinking. The legal limit in Canada, Austria, and the United Kingdom is 0.08 percent. Legal limits in Australia range from 0.05 to 0.08 percent. The Netherlands, Finland, France, Germany, and Japan all have 0.05-percent legal limits. Sweden has lowered the legal BAC limit for drivers to 0.02 percent (NHTSA 1997).

## Legislative Measures

Society has implemented a variety of legislative measures to reduce alcohol-impaired driving. To date, U.S. laws have not attempted to restrict adult drivers to moderate drinking. Nonetheless, research indicates that laws adopted in the United States have reduced alcohol-related traffic deaths among both moderate drinkers and those who have very high blood alcohol levels. Current U.S. laws that limit drivers’ BACs to 0.10 and 0.08 percent have been found effective for reducing alcohol-related crashes, as have license revocation/suspension and other penalties for exceeding those limits as well as laws restricting access to alcohol.

### Legal BAC Limits for Drivers

Reducing the BAC level at which people can legally drive can effectively reduce alcohol-related traffic crashes (e.g., Johnson and Fell 1995; Hingson et al. 1996). Drivers under age 21 are subject to lower BAC limits than older drivers, and a few States have set lower limits for drivers previously convicted of driving while intoxicated (DWI).

#### Lowering the Legal BAC Limits to 0.08 Percent for Drivers Over Age 21

Seventeen States have lowered the criminal per se legal BAC limit from 0.10 to 0.08 percent for noncommercial drivers age 21 and older (lower limits have been established for drivers younger than age 21 and are discussed later in this article). According to the criminal per se provision, prosecutors are not required to introduce evidence other than a BAC of 0.08 percent or higher to demonstrate impairment, thereby making convictions easier to obtain. These laws also have administrative license revocation (ALR) provisions, which permit police officers to immediately seize the license of any driver with a BAC of 0.08 percent or higher. The person’s license is then suspended until either a hearing or trial is conducted.

Several studies have examined the effects of 0.08 laws on fatal crash trends. In California, the largest State to adopt a 0.08 law, researchers found a 12-percent decline in alcohol-related fatal crashes after the law was adopted. Because California also adopted an ALR law 6 months after the criminal per se law, the separate effects of each law are difficult to determine (NHTSA 1991). According to one study, most of the effects occurred after the ALR provisions were added (Rogers 1995).

Johnson and Fell (1995) monitored six measures of driver alcohol involvement in the first five States to adopt 0.08 laws (Utah, Oregon, Maine, California, and Vermont) and identified several statistically significant pre-to post-law decreases. Because the study did not compare States with the 0.08 law with States that did not have the law, researchers could not determine whether the changes were independent of general regional trends. The researchers concluded that the effects of the law were independent of national trends.

**Figure 2:**
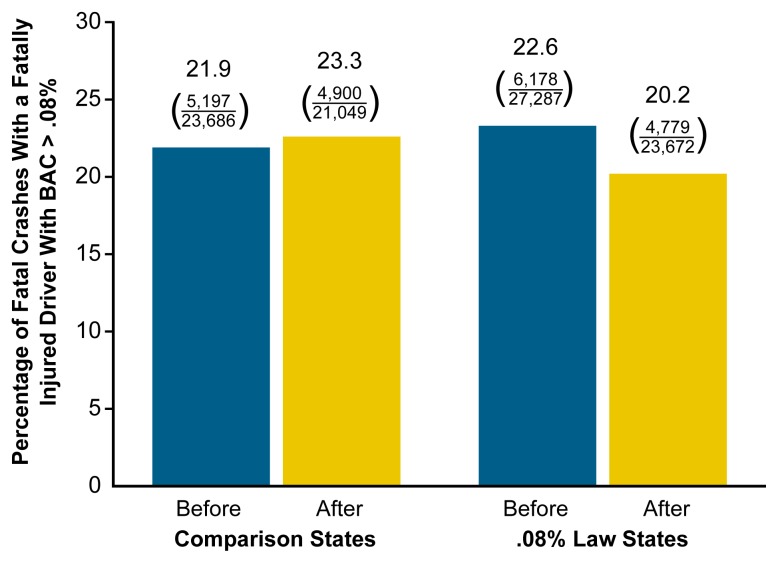
Proportion of fatal crashes with fatally injured drivers who had blood alcohol concentrations (BACs) of .08% or higher before and after the implementation of laws reducing the legal BAC for driving to .08%. Researchers compared States that had BAC limits of .10% (i.e., comparison States) with States that passed laws making it illegal to drive with BACs greater than .08% (i.e., .08% law States). Over the period of the study, the proportion of fatal crashes with fatally injured drivers who had BACs of .08% or higher increased slightly in the comparison States but declined in the .08% law States, indicating that the .08% laws worked to reduce the occurrence of this type of fatal crash. SOURCE: Hingson, Heeren, and Winter 1996.

In a subsequent analysis, Hingson and colleagues (1996) paired the aforementioned first five States to adopt a 0.08 law with five nearby States that retained the 0.10-percent legal BAC limit (Idaho, Washington, Massachusetts, Texas, and New Hampshire). Relative to the comparison States, the States that adopted the 0.08 law experienced a significant reduction (i.e., 16 percent) in the proportion of fatal crashes involving fatally injured drivers with BACs of 0.08 percent or higher (see figure 2). These results resembled those reported in both the United Kingdom and France when those countries first combined 0.08 laws with automatic license revocation provisions (Ross 1973; Ross 1982).

The majority of drivers killed in traffic crashes in 1997 who tested positive for alcohol had BACs of 0.15 percent or higher. The first five States to adopt the 0.08 law also experienced a significant decline (i.e., 18 percent) relative to the comparison States in the proportion of fatal crashes involving fatally injured drivers with BACs of 0.15 percent or higher (Hingson et al. 1996). Compared with 0.10-percent States, the 0.08-percent States may have been more concerned about alcohol-impaired driving and more responsive to legislative initiatives to reduce the problem. For example, all five 0.08 States had ALR laws during the study period, and three of the States implemented ALR laws within 1 year of the 0.08 law. This proximity restricted the study’s ability to separate the effect of the 0.08 laws from that of the ALR laws. ALR laws alone have been associated with a 5-percent decline in both alcohol- and non-alcohol-related fatal crashes and as much as a 9-percent decline in alcohol-related fatal crashes (Zador et al. 1989).

In another study, researchers paired six additional States that had adopted 0.08 laws in 1993 and 1994 (Kansas, North Carolina, Florida, New Hampshire, New Mexico, and Virginia) with six similar contiguous or nearby States that retained a 0.10-percent legal limit (Oklahoma, Tennessee, Maryland, Georgia, Colorado, and Connecticut) (Hingson et al. in press). As a group, the 0.08 States experienced a 16-percent reduction in the proportion of alcohol-related fatal crashes (i.e., crashes in which either a driver or pedestrian had a BAC of 0.10 percent or higher), which was significantly greater than the 11-percent decline identified in comparison States during the same time period. Similarly, the 0.08 States experienced a 20-percent decline in the proportion of drivers with BACs of 0.10 percent or higher who were involved in fatal crashes. This decline was significantly greater than the 14-percent decline in the comparison States. Declines experienced by comparison States were not significantly different from those experienced by other 0.10 States across the country during the same time period. Four of the 0.08 States (i.e., Kansas, North Carolina, Florida, and New Mexico) had ALR laws in place before the study period. Therefore, ALR laws in those States could not confound the study findings. Relative to the comparison states, the four 0.08 States with ALR laws experienced a significantly greater decline in the proportion of alcohol-related fatal crashes (17 versus 13 percent) and in the proportion of drivers involved in fatal crashes with BACs of 0.10 percent or higher (21 versus 16 percent). Thus, independent 0.08 law effects were seen in these States, although the effects were smaller than the effects seen in studies of States that adopted both 0.08 and ALR laws simultaneously or within a close timeframe (Hingson 1996; Rogers 1995).

In the most comprehensive study of its type to date, a national study conducted over a 16-year period identified an 8-percent decline in the proportion of drivers with positive BACs involved in fatal crashes in States that had adopted 0.08 laws. This reduction was independent of the effects of other DWI laws, such as the 0.10-percent BAC limit and ALR laws. The study also controlled for the effects of safety belt laws and potentially confounding trends in demographic, economic, and seasonal factors and alcohol consumption. The reduction was observed among both drivers with BACs from 0.01 to 0.09 percent and drivers with BACs of 0.10 percent and higher (Voas and Tippets 1999). In addition, an 11-State study found that 0.08 legislation, either alone or in conjunction with ALR laws, was associated with declines in alcohol-related fatalities in seven States, and significant declines were associated specifically with 0.08 laws in five States (Apsler et al. 1999). It appears that 0.08 percent laws have effects independent of ALR laws but that their greatest impact is in combination with ALR laws.

For legal BAC limits to most effectively deter alcohol-impaired driving, the public must be informed of them. A national survey of more than 4,000 drivers (NHTSA 1996) found that only 54 percent of residents in States with 0.08 laws and only 38 percent of residents in States with 0.10 laws could correctly identify their State’s BAC limit. When the legal BAC limits for drivers are lowered, the need to educate the public about these changes is apparent. Research indicates that public education to promote awareness of a State’s new legal BAC limit can enhance the legislation’s effects (Blomberg 1992).

Public education also is needed to increase drivers’ knowledge about the impairments associated with different levels of alcohol consumption. Among drivers surveyed, 75 percent thought that at least one-half of all drivers would be dangerous if they drove after consuming five drinks in 2 hours, but only 28 percent of the respondents thought all drivers would be unsafe under those conditions (NHTSA 1996).

#### Zero-Tolerance Laws for Drivers Under Age 21

Although all States prohibit people under age 21 from purchasing, possessing, or consuming alcohol, drinking remains prevalent among teenagers (Johnston et al. 1998). Zero-tolerance laws are designed to reduce drinking and driving among young people by making it illegal for persons under 21 to drive after any drinking. These laws set the legal BAC limit for drivers under age 21 at 0.00 or 0.02 percent. In the fall of 1995, the U.S. Congress mandated that Federal highway funds be withheld from States that did not adopt zero-tolerance laws. At that time, only one-half of the States had such laws.

**Figure 3:**
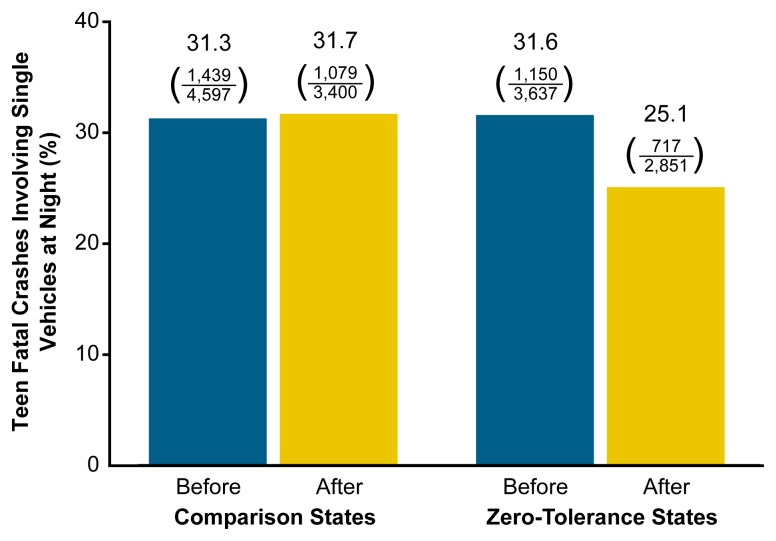
Proportion of teen fatal crashes involving single vehicles at night before and after zero-tolerance laws for youth. Researchers compared States that had passed laws making it illegal for underage youth to drive with a measurable blood alcohol concentration (BAC) (i.e., zero-tolerance States) with States that had not passed such legislation (i.e., comparison States). Over the period of the study, the proportion of teen fatal crashes involving single vehicles at night increased slightly in the comparison States but declined in the zero-tolerance States, indicating that the zero-tolerance laws worked to reduce the occurrence of this type of fatal crash. SOURCE: Hingson, Heeren, and Winter 1994.

A recent study (Hingson et al. 1994) found that the first eight States to adopt zero-tolerance laws experienced a 20-percent greater decline in the proportion of nighttime single-vehicle fatal crashes among 15- to 20-year-old drivers compared with eight nearby States without zero-tolerance laws (see figure 3). Single-vehicle nighttime crashes are the type of fatal crash most likely to involve alcohol.

States that did not adopt zero-tolerance laws but lowered their BAC limits for drivers under age 21 to either 0.04 or 0.06 percent experienced declines of 6 to 7 percent relative to States with no BAC limit specific to drivers under age 21 (Hingson et al. 1994). Setting BAC limits for young drivers at 0.04 or 0.06 percent allows youth to speculate about how much they can drink and still drive legally. Conversely, zero-tolerance laws send a clear message to young drivers that it is illegal to drive after engaging in *any* drinking. By the summer of 1998, all 50 States had passed zero-tolerance laws.

Unfortunately, some States have experienced difficulty in achieving broad public awareness of zero-tolerance laws. Studies in both California and Massachusetts found that 45 to 50 percent of young drivers were unaware of the law (Martin et al. 1996). Obviously, increasing awareness of the zero-tolerance law can enhance its effects. Blomberg (1992) found a one-third greater decline in alcohol-involved crashes among young drivers in Maryland counties where public service announcements about the State’s zero-tolerance law were aired compared with drivers in other counties.

**Figure 4:**
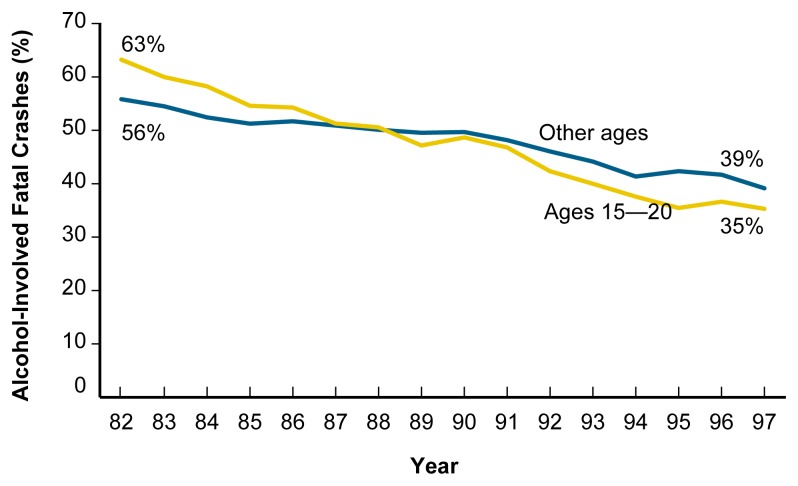
Proportion of fatal crashes that involved alcohol, 1982 to 1997. Since 1982 the proportion of fatal crashes that involve alcohol has steadily declined. SOURCE: National Highway Traffic Safety Administration 1998*a*.

As a result of all States raising the minimum legal drinking age (MLDA) to 21 and adopting zero-tolerance laws for persons under age 21, the greatest reductions in alcohol-related traffic crashes in recent years have occurred among drivers under 21. Since NHTSA first began conducting national estimates of alcohol involvement in fatal crashes, alcohol-related fatalities among 15- to 20-year-olds have declined 59 percent, from 5,380 in 1982 to 2,209 in 1997. By comparison, alcohol-related fatalities among all other age groups declined 29 percent, from 19,785 to 13,980 (NHTSA 1998*a*) (see figure 4). The proportion of all fatal crashes among 15- to 20-year-olds that involved alcohol declined 44 percent compared with 30 percent for all other age groups from 1982 to 1997.

#### Lower Legal BAC Limits for Drivers Convicted of Driving While Intoxicated

Similar to drivers under age 21, drivers with prior DWI convictions are disproportionately at risk for alcohol-related fatal crash involvement. One study found that drivers killed in alcohol-related crashes were eight times more likely to have had a DWI conviction in the previous 5 years than drivers randomly selected from the general population of licensed drivers (Brewer et al. 1994).

In August 1988 Maine lowered the legal BAC limit for drivers previously convicted of DWI to 0.05 percent. In 1995 the law was modified to make it illegal for these drivers to drive after engaging in *any* drinking. The law allows first-time DWI offenders’ licenses to be reinstated after a mandatory suspension of 2 months on the condition that they not drive with a positive BAC for 1 year. Licenses of second-time offenders are reinstated on the condition that the offenders not drive with a positive BAC for 10 years. Convicted DWI offenders who are apprehended with positive BACs receive a 1-year administrative license suspension or revocation and may receive court-imposed penalties.

One method for measuring the effectiveness of laws lowering BAC limits for DWI-convicted drivers is to determine any pre- to post-law changes in the extent to which DWI offenders are involved in fatal crashes. During the first 6 years after Maine adopted its 0.05-percent BAC limit for drivers with prior DWI convictions, the proportion of fatal crashes involving drivers with prior DWI convictions declined 25 percent, compared with the 6 years before the law took effect. The proportion of fatal crashes involving drivers with prior DWI convictions and BACs of 0.05 percent or higher declined 31 percent (Hingson et al. 1998).

Opponents of lowering the legal BAC limits for drivers have argued that these measures target “social drinkers” and have no effect on drivers with high BACs or prior DWI convictions. However, during the 6 years after Maine adopted its 0.05-percent law for drivers with prior DWI convictions, the proportion of fatal crashes involving drivers with prior DWI convictions and BACs of 0.15 percent or higher declined 35 percent. During those 6 years, compared with the previous 6 years, the rest of the United States experienced minimal change in the proportion of fatal crashes involving drivers with prior DWI convictions and positive BACs. In the rest of New England, the proportion of fatal crashes involving drivers with prior DWI convictions and positive BACs increased over 40 percent (Hingson et al. 1998).

In 1995 Maine became the first State to adopt a zero-tolerance law for convicted DWI offenders. Maine was also the first State to adopt a zero-tolerance law for drivers under age 21 in 1983. Because of the benefits of that law, by the end of 1998, all States had adopted a zero-tolerance law for drivers under 21. Further research is needed to evaluate the effects of Maine’s second zero-tolerance law.

### Sanctions for Drivers Convicted of DWI

Other measures to reduce recidivism among persons convicted of DWI include such sanctions as jail sentences, mandatory alcoholism treatment, and license plate or vehicle impoundment. In a meta-analysis of over 200 studies, Wells-Parker and colleagues (1995) reported that alcoholism treatment was associated with a 7- to 9-percent reduction in alcohol-related crashes compared with standard sanctions (e.g., jail or fines). Sanctions combining punishment, education, and therapy with monitoring and aftercare were more effective for both first-time and repeat offenders than any single approach.

Various studies also have found that impounding of DWI offenders’ vehicles or license plates reduces recidivism (Voas et al. 1997, 1998; Rodgers 1994), as does the use of ignition interlock devices that prevent vehicle operation when a driver’s breath alcohol exceeds a designated limit (Beck et al. in press).

### Restricting Access to Alcohol

Another approach to reducing drinking and driving is to lower the accessibility of alcohol. Decreased accessibility can be accomplished by raising the price of alcohol through increased taxes, restricting both alcohol outlet density (i.e., the number of alcohol outlets in an area) and hours of operation, maintaining State control of alcohol sales, and implementing laws to restrict alcohol sales to inebriated persons or persons under age 21 (Kenkel and Manning 1996; Gruenewald et al. 1996; DeJong and Hingson 1998). Research shows that alcohol-related traffic fatalities can be reduced by increasing taxes on alcohol (Cook 1981; Saffer and Grossman 1987*a*; 1987*b*; Grossman et al. 1991) and enforcing laws that hold alcohol servers responsible for actions taken by under-age persons or intoxicated patrons who were sold alcohol (McKnight 1993; McKnight and Streff 1994; Wagenaar and Holder 1991*b*; Sloan et al. 1994). The operation of alcohol outlets by private owners rather than government agencies has been associated with increased alcohol consumption (Gruenewald et al. 1993; Gruenewald and Ponicki 1995; Wagenaar and Holder 1991*a*; Holder and Wagenaar 1990), and increased outlet density has been associated with increased alcohol-related traffic fatalities (Scribner et al. 1994).

#### Minimum Legal Drinking Age

Raising the MLDA to 21 was designed to reduce impaired driving by restricting the accessibility of alcohol to everyone under age 21. This law tried to eliminate even moderate drinking by adolescent drivers. In 1984, when the Federal Government passed the National Minimum Drinking Age Act, 25 States had MLDAs of 21. By 1988 all 50 States had adopted MLDAs of 21.

**Figure 5:**
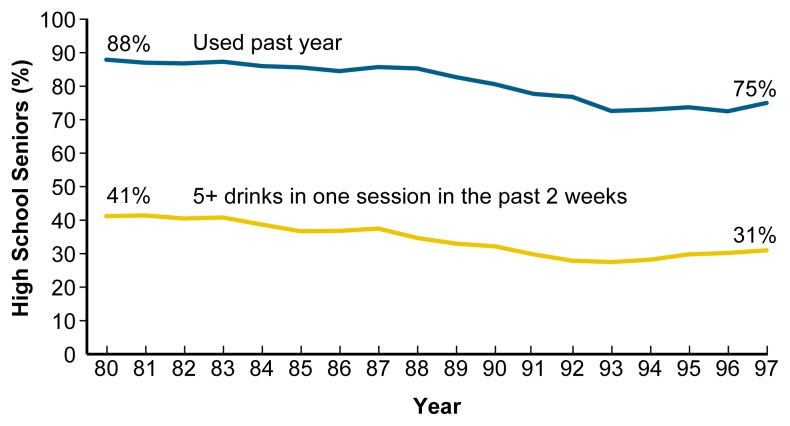
Alcohol use by high school seniors. Since 1980 high school seniors’ use of alcohol has steadily declined. SOURCE: Johnston et al. 1998.

Survey results show a decline in drinking among young people following the increase in the MLDA. The proportion of high school seniors who reported drinking during the year before being surveyed declined from 88 percent in 1980 to 75 percent in 1997. The proportion of high-school seniors who reported consuming five or more drinks on at least one occasion in the past 2 weeks declined from 41 to 31 percent (Johnston et al. 1998) (see figure 5). In addition to the decrease in drinking observed among persons under age 21 following the increase in the MLDA, research also suggests that raising the MLDA resulted in reduced drinking among 21- to 25-year-olds who grew up in States with a MLDA of 21 compared with those who grew up in other States (O’Malley and Wagenaar 1991).

**Figure 6:**
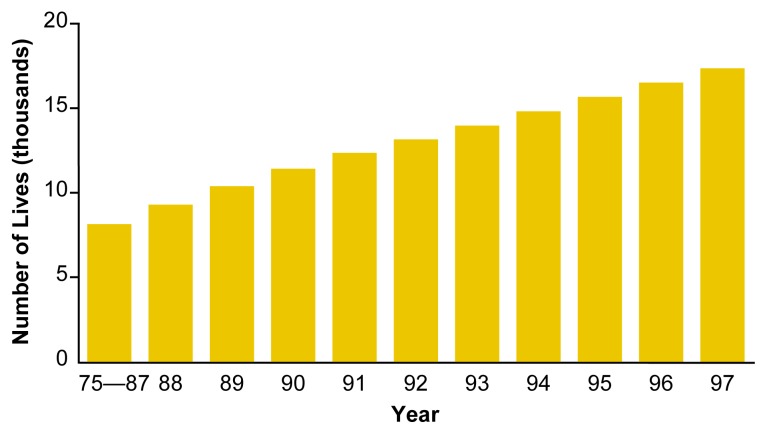
Cumulative estimated number of lives saved by the minimum drinking age laws, 1975 to 1997. The minimum drinking age laws are credited with having saved increasing numbers of lives among the general U.S. population since 1975. SOURCE: National Highway Traffic Safety Administration 1998*b*.

Numerous studies have indicated that raising the MLDA to 21 reduces alcohol-related fatal crash involvement among drivers under age 21 (United States General Accounting Office 1987). Of the 29 studies completed since the early 1980s that evaluated increases in the MLDA, 20 studies showed significant decreases in traffic crashes and crash fatalities for persons under age 21. Only three studies found no change in traffic crashes involving youth. The remaining six studies had equivocal results (Toomey et al. 1996). States adopting MLDAs of 21 in the early 1980s experienced a 10- to 15-percent decline in alcohol-related traffic deaths among young drivers compared with States that did not adopt such laws. NHTSA (1998*b*) has estimated that the raising of the MLDA to 21 has prevented more than 17,300 traffic deaths among persons under age 21 since 1975, approximately 700 to 1,000 deaths annually for the past decade (see figure 6). Research has not examined whether MLDA laws have also reduced alcohol-related crash involvement among 21- to 25-year-olds who grew up in States with a MLDA of 21 relative to those who grew up in other States.

Despite the declines in teenage drinking and fatal crashes associated with prohibiting the purchase and possession of alcohol by persons under age 21, underage youth throughout the United States still can obtain alcohol easily from many sources. Heightened enforcement of MLDA laws can reduce youth access to alcohol, however. Preusser and colleagues (1994) reported that alcohol sales to underage youth declined dramatically following an enforcement campaign targeted at retail alcohol vendors. The campaign involved four “sting operations” over 10 months in which under-age male police cadets attempted to purchase alcohol at liquor, grocery, and drug stores. Store owners received warnings if cadets were able to purchase alcohol on their first attempt. Stores that sold alcohol to the cadets during subsequent attempts were penalized accordingly. Over the 10-month period the cadets’ rate of successful purchase declined from 59 to 26 percent.

## Conclusion

Although moderate drinking can impair driving performance, this level of alcohol consumption would not cause a person’s BAC to reach the legal limit for most drivers in the United States. In most States, the legal BAC limit for drivers age 21 and older is 0.10 percent. Based on this standard, the public may incorrectly assume that a driver is not impaired at BACs lower than 0.10 percent. In one study, only 28 percent of the drivers surveyed reportedly believed that all drivers would be unsafe after consuming amounts of alcohol that would increase a person’s BAC to 0.08 percent. However, virtually all drivers are seriously impaired at this BAC level. Drivers must become more informed about alcohol consumption’s effects on BAC levels and the various impairments a person experiences as a result of increasing BAC levels. Educational programs about alcohol consumption and impairment must be developed, implemented, and evaluated in an effort to reduce alcohol-related traffic crashes.

Research has demonstrated the effectiveness of lowering the legal BAC limit to 0.08 percent for drivers over age 21 and to 0.05 percent for drivers with prior DWI convictions, as well as implementing zero-tolerance laws for drivers under age 21. Further research is needed to determine whether Maine’s zero-tolerance law for drivers with prior DWI convictions will further reduce their involvement in fatal crashes just as zero-tolerance laws did for drivers under age 21. The decline in alcohol-related fatal crashes associated with Maine’s 0.05 law for convicted DWI offenders suggests that it warrants consideration and study in other States as well. Increased knowledge of what constitutes moderate drinking and the amount of alcohol a person can reasonably consume before becoming impaired would help raise people’s sense of responsibility, both as drinkers and as drivers, ultimately saving thousands of lives.
